# 

**Published:** 1863-12

**Authors:** 


### Editorial.

### CLOSE OF THE VOLUME.

This number closes the 17th volume of the REGISTER. In regard to the course pursued in the conduct of the Register, it is scarcely necessary to say anything.

We have simply aimed to pursue, so far as circumstances would permit, such a course as would result in the greatest good to the profession. Various circumstances have prevented its prompt issue at the beginning of each month; but we hope, in the future, we will not be pressed with so much and such a variety of labor. We are again invested with the ownership of the Register; and hope we shall have the aid of our brethren to make it what is demanded by the profession. It will be issued monthly, and the same size as heretofore.

With this number we send out bills to the few of our subscribers who are still in arrears for volume 17, and we desire to hear from them all immediately; and we hope all our subscribers will remember that the subscriptions are payable in advance as far as possible. We are determined not to make a large list of delinquent or non-paying subscribers. The edition issued will be regulated by the state of the subscription list; therefore we hope that all who intend to subscribe will notify us of the fact as early as possible. T.

YPSILANTI, Jan. 1, 1864.

*Dear Sir:*—The Annual Meeting of the MICHIGAN DENTAL ASSOCIATION, will be held in the city of Jackson, on Tuesday, January 19th, 1864, at 9 o'clock A. M.

The invitation is intended to be general; and it is hoped that the profession will turn out and give their hearty support to the Association, which is so essentially necessary at the present time. Very respectfully Yours,

J. A. WATLING, *Sec'y.*

### DENTAL REGISTER
### OF THE WEST,

ISSUED MONTHLY, AT $3 A YEAR IN ADVANCE.

DEVOTED TO THE INTERESTS OF THE DENTAL PROFESSION.

EDITED BY J. TAFT AND GEO. WATT.

The Volume commences in January and closes in December.

The Register being issued monthly is always fresh, therefore indispensable to the practitioner who desires to keep posted up with the new inventions and improvements which are daily developed. Neither expense nor effort will be spared to give value and variety to its contents. Articles will be illustrated with well executed engravings. whenever they will add to the interest or assist in explanation.

Its extensive circulation and frequency of issue makes it one of the BEST DENTAL ADVERTISING MEDIUMS in the United States.

RATES OF ADVERTISING.

1 page, 1 year, $50. ½ page, 1 year, $30. ¼ page, or card, 1 year, $20.

All advertising bills payable in advance, or at furthest after the issue of the first uumber containing the advertisement. All transient advertisements to be paid for in advance.

☞ CONTRIBUTIONS SOLICITED.

Address Original Communications to **Geo. Watt, Xenia, o.**
Exchanges to **J. Taft, or Dental Register, Cincinnati, Ohio.**
Business Communications to

J. TAFT, Publisher,
172 Race Stree.

